# Male-female differences in 6-sulfatoxymelatonin excretion in hypopituitary patients

**DOI:** 10.1590/2359-3997000000153

**Published:** 2015-01-01

**Authors:** Hugo L. Fideleff, Gabriel Fideleff, Hugo R. Boquete, Martha Suárez, Miriam Azaretzky

**Affiliations:** 1 Unidad de Endocrinología Departamento de Medicina Hospital T. Alvarez Buenos Aires Argentina Unidad de Endocrinología, Departamento de Medicina, Hospital T. Alvarez, Buenos Aires, Argentina

**Keywords:** 6-sulphatoxymelatonin, melatonin and GH, adult GH deficiency, growth hormone treatment, melatonin and sexual dimorphism

## Abstract

**Objective:**

To evaluate melatonin secretion in adult hypopituitary patients with Growth Hormone deficiency (AGHD) on and off replacement therapy.

**Subjects and methods:**

We studied 48 subjects: 12 (6 males) untreated AGHD (AGHDnt), 20 (10 males) treated AGHD (AGHDt) and 16 healthy subjects (8 males) as control group (CG). We measured urinary 6-sulfatoxymelatonin (6-SM) in total (24 h samples), nocturnal (6-SMn): 1800-0800 and diurnal samples (6-SMd): 0800-1800.

**Results:**

Significant differences were observed among the 3 groups of male subjects, in total 6-SM (p < 0.05), nocturnal 6-SM (p < 0.02) and nighttime-daytime delta values (p < 0.003). CG had significantly higher values than the AGHDnt in total 6-SM (p < 0.01), nocturnal 6-SM (p < 0.05) and nighttime-daytime delta values (p < 0.01). AGHDt patients showed significantly higher levels in nighttime-daytime delta values than AGHDnt patients (p < 0.05). In females, no significant differences were found among the 3 groups studied in total, nocturnal, diurnal or nighttime-daytime delta values. In males, significant correlations were found among total 6-SM (r = 0.58; p = 0.029), nocturnal 6-SM (r = 0.70; p = 0.006) and nighttime-daytime delta values (r = 0.71; p = 0.004) vs. serum IGF-1 levels in subjects evaluated. In females, significant correlations were found among total 6-SM (r = 0.57; p = 0.02) vs. serum IGF-1 levels in subjects evaluated. A tendency towards a significant correlation was found in diurnal 6-SM (r = 0.48; p = 0.07).

**Conclusions:**

Our findings show a sexual dimorphism in 6-SM excretion in AGHD patients and provide an interesting approach to a further understanding of some chronobiological disorders involved in GH deficiency.

## INTRODUCTION

Melatonin acts as a “neuroendocrine transducer” of environmental information collected through a retina-pineal gland neuronal circuit in which other structures are involved, including but not limited to, the retino-hypothalamic tract, the supra-chiasmatic nucleus and the cervical ganglion. The relationships between melatonin and different pituitary hormones and sex steroids have been extensively studied; however, the relationship between growth hormone (GH) and melatonin remains unclear (1-3). Pineal gland can influence GH-IGF-1 function and, in mechanism of this dependence, changes in endogenous melatonin concentrations seem to develop an important role.

Many years ago, melatonin has been reported to induce an increase in GH levels in human subjects (4,5); however, other groups failed to show the effects of melatonin on GH secretion (6,7). In addition, *in vitro* experiments showed that melatonin reduced GH secretion from rat pituitary cells (8). These variations in the relationship between melatonin and GH might be due to multiple factors, such as differences in the experimental models, physiological conditions, different actions depending on the species studied, etc. (9). GH is released largely at night in complex ultradian pulses that do not readily fit a classic circadian pattern. Indirectly, the clock system directs secretion through slow-wave sleep, which is strongly associated with GH release, although the precise mechanism of circadian influence is not understood (10). We have demonstrated in a recent paper decreased 6-sulphatoxymelatonin (6-SM) excretion in male GH-deficient children and adolescents (11). Taking into account that there is sexual dimorphism of plasma GH with distinct male and female patterns of growth, which may be clock-determined (1), and considering that melatonin is a key hormone in the biological clock and in the day/night cycle, we decided to evaluate melatonin secretion in a group of GH-deficient male and female adults on and off replacement therapy, by measuring the excretion of its major urinary metabolite 6-SM (12,13). To evaluate possible alterations in the circadian rhythm of melatonin secretion in this group of patients, we decided to perform not only 24 hour urinary measurements, but also measurements in diurnal and nocturnal samples and to calculate nighttime-daytime delta values.

## SUBJECTS AND METHODS

In a prospective not paired study, we evaluated 48 subjects, divided into 12 untreated GH-deficient adults (AGHDnt) (19-44 yr, 6 males), 20 treated GH-deficient adults (AGHDt) (21-56 yr, 10 males), and 16 healthy subjects (27-65 yr, 8 males) as control group (ACG). Patients were selected at random from a cohort of hypopituitary patients followed at the out clinic of our Unit, while controls were healthy members of the staff of the unit and volunteers. Baseline patient characteristics are depicted in Table 1. In the AGHDnt group, 5 were idiopathic, 2 pituitary hypoplasia, 2 post surgery of prolactinoma, 2 perinatal anoxia and 1 post surgery of Cushing disease. As regards the AGHDt group, 6 were idiopathic, 4 postsurgery of non functioning pituitary adenoma, 3 Sheehan syndrome, 3 pituitary hypoplasia and 1 perinatal anoxia, postencephalitis, empty sella and post surgery of prolactinoma each. GH deficiency was confirmed by an insulin tolerance test with GH response of < 3 ng/mL and/or arginine stimulation test < 1.4 ng/mL. Patients were treated during a period of 9 months to 4 years with GH doses to maintain IGF-1 between 0 and +2 SDS. All patients maintained stable doses for at least 6 months. Patients with other associated hormonal deficits were receiving adequate replacement. In the AGHDnt group, 11/12 were gonadotropin deficients and all were substituted; in the AGHDt group 18/20 were gonadotropin deficients and 13 of them were under sex steroids replacement. Of the 5 patients not substituted, one was a male with benign prostatic hypertrophy and 4 women were menopausal. Near the time the urinary sampling, in TSH deficient patients, during replacement therapy, mean fT4 levels were 1.16 µg/dL, while in gonadotropin deficient patients, during replacement therapy, mean testosterone levels were 4.2 ng/mL for men and estradiol levels were 51 pg/mL for women receiving estradiol valerate (for those receiving CEE, there were no estradiol measurements).

**Table 1:** Baseline patient characteristics

	AGHDnt n: 12	AGHDt n: 20	ACG n: 16
Chronological age range (yr)	19-44	21-56	27-65
Gender (m/f)	6/6	10/10	8/8
BMI (kg/m^2^) (mean and range)	26.32 (18-38.5)	25.82 (19-40)	23.8 (21.1-28.8)
Organic GH deficiency	7/12	14/20	–
Idiopathic GH deficiency	5/12	6/20	–
Isolated GH deficiency	0/12	2/20	–
Panhypopituitarism (GH, ACTH, TSH, LH/FSH)	9/12	14/20	–
GH, TSH and LH/FSH deficiency	2/12	4/20	–
GH and TSH deficiency	1/12	0/20	–

AGHDnt: adult GH deficient non-treated patients; AGHDt: adult GH deficient treated patients; ACG: adult control group.

Patients with craniopharyngioma were excluded. Normal subjects were healthy volunteers. Subjects with any clinical or endocrine pathology or those receiving medication were excluded from the sample. Both patients and healthy controls showed no clinical evidence of sleep apnea or any other sleep disorder, evaluated through an exhaustive questioning of patients and/or his/her partner and all had normal liver function tests. They lived in Buenos Aires or its surroundings (34º37’S, 58º25’W). All serum samples were collected during spring time. Informed consent was obtained from all subjects. The study was conducted according to the Decla ration of Helsinski II and the Guidelines for Good Clinical Practice. The protocol was approved by the Ethical and Research Committees of the participating centers.

### Urine collection

In all patients and controls a 24-hr urine sample was collected at home during two intervals: (i) a 14-hr nocturnal sample (from 1800 to 0800 h), and (ii) a 10-h diurnal sample (from 0800 to 1800 h). Detailed verbal and written instructions were given to all the subjects to assure complete collection of samples. All collections were made on Sunday to avoid possible interference with occupational activities. All patients were instructed to maintain their usual diurnal activities, as well as the characteristics, time and duration of sleep and to keep a dark environment. Collected urines were stored in a refrigerator until delivered to the laboratory within 24 h of urine collection. The volume of each urine collection was measured and aliquots were put in plastic bottles without preservatives and stored frozen (-70ºC) until assayed. A trained laboratory technician was responsible for receiving the samples and checking them for completeness. Urine samples with volumes lower than expected were discarded.

### Laboratory measurements

Blinded analysis of urine 6-sulfatoxymelatonin levels was performed by radioimmunoassay using an assay kit from Stockgrand Ltd. (Guildford, UK) as previously described (12). The urine samples were diluted prior to assay (1/250). The intra- and interassay coefficients of variation (CV’s) were 4% and 7%, respectively. Excretion of 6-sulfatoxymelatonin was expressed as: (i) total amount excreted (µg), (ii) µg excreted per time interval and (iii) estimated amplitude: the difference between nocturnal and diurnal samples.

Sample collection for IGF-1 and stimulation tests for diagnosis of GH deficiency were performed at 0800 under fasting conditions. GH was measured by a solid phase*, *2*-*site chemiluminescent enzyme immunometric assay (IMMULITE^®^ 2000), calibrated against the first reference standard (WHO International Reference Preparation 80/505); as from batch 206 calibrated against the second reference standard (WHO International Reference Preparation 98/574). The detection limit was 0.08 ng/mL and the intra- and interassay CVs were below 3% for a dose of 1.8 ng/mL and below 5% for a dose of 9.6 ng/mL. IGF-1 was measured by an automated chemiluminiscent assay system (IMMULITE^®^ 2000); this is a two-site, solid-phase chemiluminiscent enzyme immunometric assay. The detection limit was 20 ng/mL. Intra- and interassay CVs were below 5.1% for a dose of 59 ng/mL and below 6.5% for a dose of 230 ng/mL. The standard was calibrated against the WHO International Reference Preparation 87/518. IGF-1 values were expressed in terms of SDS allowing comparing patients of different ages. The SDS for IGF-1 was calculated according to our laboratory reference values.

### Statistical analysis

Total, diurnal, nocturnal 6-SM levels and nighttime-daytime differences (delta values), as well as age and BMI were compared among the different subgroups by the Kruskal-Wallis, Dunn test. To compare GH doses and IGF-1 levels between males and females GH treated patients a Mann-Whitney test was employed. Since only 5 patients with gonadotropin deficiency were not receiving exogenous testosterone or estrogens, we didn’t separate 6-SM levels between sex steroids treated and not treated patients.

Levels of 6-SM were correlated with IGF-1 levels in GH deficient patients using the Spearman test adjusting the lineal model with the least squares method. SPSS software, version 17.0 (SPSS Inc., Chicago, IL, USA) was employed. Results were expressed as median and range; p-values < 0.05 were considered evidence for statistical significance.

## RESULTS

There were no statistical differences in age or BMI among the three evaluated groups.

GH doses ranged: males 0.003-0.005 mg/kg/day (mean 0.004 mg/kg/day), females 0.005-0.009 mg/kg/day (mean 0.008 mg/kg/day), p = 0.0005 males *vs* females. Mean IGF-1 levels at the time of the urinary sampling in the untreated and treated groups were 29.6 ng/mL (SDS -6.3) and 169 ng/mL (SDS 0.8) respectively. Regarding the treated group, IGF-1 SDS levels were in males 0 – 1.8 (mean 0.9) and females 0 – 1.7 (mean 0.8), p not significant males vs females.

Total, nocturnal and diurnal 6-SM levels and nighttime-daytime delta values in AGHDnt, AGHDt and ACG in both genders are shown in Table 2.

**Table 2:** 6-SM excretion in AGHDnt, AGHDt and ACG in both genders

	Males				Females		
	AGHDnt	AGHDt	ACG		AGHDnt	AGHDt	ACG
Total 6-SM (µg/24 hr)	1.36 (0.63 – 3.25)	2.29 (0.39 – 17.0)	4.30* (1.74 – 15.9)		2.46 (0.80 – 5.70)	6.25 (0.38 – 19.0)	4.66 (0.78 – 19.2)
Nocturnal 6-SM (µg) 1800 to 0800 h	0.59 (0.38 – 1.52)	1.96 (0.21 – 16.5)	3.74** (1.28 – 14.9)		1.40 (0.49 – 5.46)	3.81 (0.29 – 18.0)	3.84 (0.40 – 18.1)
Diurnal 6-SM (µg) 0800 to 1800	0.57 (0.25 – 1.73)	0.34 (0.14 – 2.23)	0.56 (0.39 – 0.96)		0.59 (0.20 – 1.60)	1.79 (0.09 – 5.70)	0.77 (0.38 – 1.00)
Delta values (µg)	-0.12 (-0.24 – 0.26)	1.10*** (0.01 – 16.0)	3.13* (0.81 – 14.0)		0.41 (-0.32 – 5.20)	1.10 (-0.90 – 16.8)	3.16 (0.03 – 17.0)

Delta values (estimated amplitude): difference between nocturnal and diurnal samples.

AGHDnt: adult GH deficient non-treated patients; AGHDt: adult GH deficient treated patients; ACG: adult control group.

Results expressed in median and range.

* p < 0.01 vs AGHDnt; ** p < 0.05 vs AGHDnt; *** p < 0.05 vs AGHDnt (Kruskall-Wallis, Dunn test).

**Figure 1:**
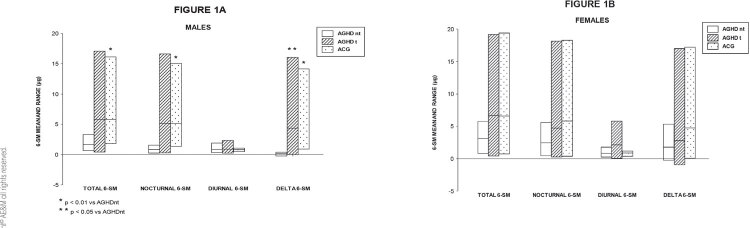
Total, nocturnal and diurnal 6-SM levels and nighttime-daytime delta values in AGHDnt, AGHDt and ACG in males (A) and females (B).

In males, significant differences were observed among the 3 groups studied, in total 6-SM (p < 0.05), nocturnal 6-SM (p < 0.02) and nighttime-daytime delta values (p < 0.003). Subsequent analysis by the Dunn test showed that ACG had significantly higher values than AGHDnt in total 6-SM (p < 0.01), nocturnal 6-SM (p < 0.05) and nighttime-daytime delta values (p < 0.01). AGHD treated patients showed significantly higher levels in nighttime-daytime delta values than AGHD untreated patients (p < 0.05) (Figure 1A).

In females, no significant differences were found among the 3 groups studied in total, nocturnal, diurnal or nighttime-daytime delta values (Figure 1B).

### 6-SM/IGF-1 correlations

**Figure 2:**
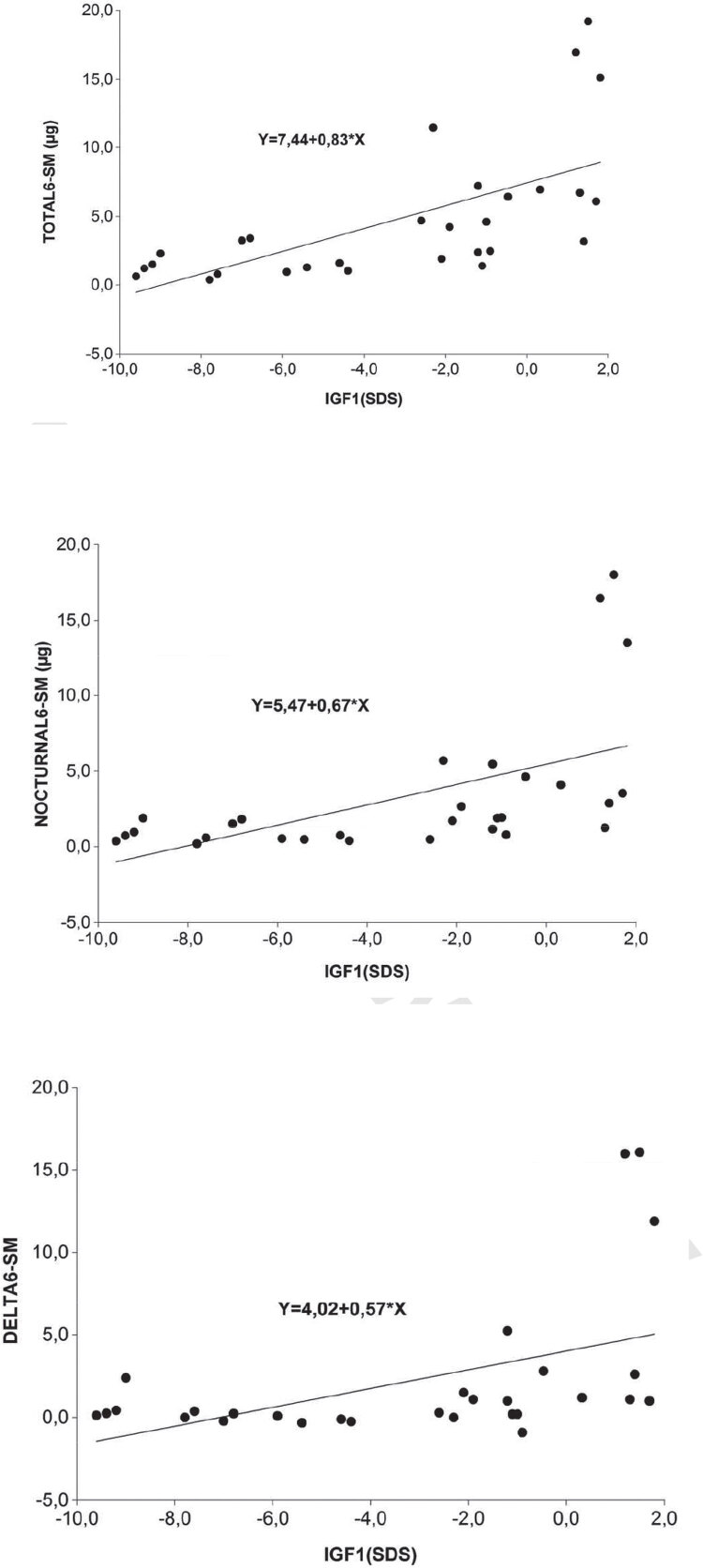
Correlations between 6-SM vs IGF-1 levels in males. (A) Total 6-SM vs IGF-1 levels. (B) Nocturnal 6-SM vs IGF-1 levels. (C) 6-SM nighttime-daytime delta values vs IGF-1 levels.

In males, significant correlations were found among total 6-SM (*R*^2^ = 0.40; R = 0.64; p = 0.0002), nocturnal 6-SM (*R*^2^ = 0.30 (R = 0.55; p = 0.0019) and nighttime-daytime delta values (*R*^2^ = 0.23 (R = 0.48; p = 0.008) *vs*. serum IGF-1 levels in subjects evaluated (Figures 2A, 2B and 2C). Correlation between diurnal 6-SM and IGF-1 was not significant in this group of patients.

**Figure 3:**
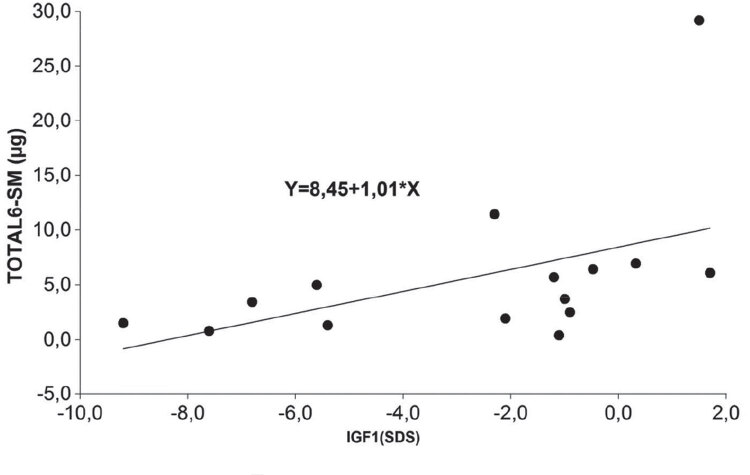
Correlations between total 6-SM vs IGF-1 levels in females.

In females, only a tendency towards a positive correlation, but no statistical significance was observed between total 6-SM *vs*. serum IGF-1 levels (*R*^2 ^= 0.23; R = 0.48; p = 0.0698) (Figure 3). Correlations between nocturnal 6-SM, diurnal 6-SM and nighttime-daytime delta values *vs*. IGF-1 were not significant in this group of patients.

## DISCUSSION

In the present study we observed, in adult males, significant differences in total, nocturnal, nighttime-daytime delta 6-SM values between controls and untreated GHD subjects. Additionally, it is worth mentioning that treated patients showed significantly higher levels of nighttime-daytime delta 6-SM than untreated GHD patients. As to women, no differences were found in 6-SM excretion among controls, untreated and treated GHD patients.

It is worth mentioning that melatonin has, in physiological conditions, a circadian secretory rhythm with low concentrations during daytime and high concentrations at night, with peak concentrations at approximately 0300 h (14). In previous studies, maximum pineal activity has been reported to occur in childhood and progressively decline in the following decades of life (14,15). Even if the most well known function of melatonin in human beings is its contribution to synchronization of biologic rhythms, with a key role in light-dark cycles, an important body of evidence in recent years has suggested that melatonin may be involved in other functions, including body temperature modulation, antiproliferative effects, antioxidant effects, interrelationship with the immune system, etc. (16-22), also acting as a “neuroendocrine transducer” of information collected from the environment (23).

The central circadian clock located in the brain influences sex hormone secretion via the hypothalamic-pituitary-gonadal axis. Because testosterone influences the GH-IGF-1 pathway, IGF-1 and its binding protein, IGFBP3, may also be influenced by circadian genes. Taken together with findings that variants in circadian genes are associated with delayed sleep phase syndrome and morning/evening preference, it is plausible that variants in these genes are associated with varying serum hormone levels (24).

Some studies indicate that the pineal gland can modify the function of GH-IGF-1 axis during the day, and these changes in endogenous melatonin concentrations seem to play an important role. Nevertheless, melatonin administration in rats after pinealectomy only partly prevented changes in GH-IGF-1 axis function caused by gland removal (3). Vriend and cols. have also shown significant increases in GH and IGF-1 concentrations performing studies on Syrian male hamsters after melatonin administration at evening hours. In the authors’ opinion, the increase in IGF-1 levels induced by melatonin administration is probably secondary to GH secretion (25). Although the relation between melatonin and hypothalamic-pituitary axis is well known, the majority of basic and clinical studies have evaluated changes in GH levels after melatonin administration or pinealectomy. To our knowledge, there is still scarce information concerning melatonin modifications after GH administration. Therefore, possible variations in melatonin in GHD patients, with and without replacement therapy, have not been adequately characterized yet. In a recent study evaluating children and adolescents, we have observed that GHD patients, both treated and untreated, showed significantly lower levels than controls in total melatonin, nocturnal levels and nighttime-daytime delta values (11).

Concerning our results, differences found in males in nocturnal melatonin levels and in nighttime-daytime delta values and the absence of differences among the three groups in diurnal melatonin secretion could lead us to speculate that GH deficiency might be involved in abnormal circadian rhythm of melatonin production. It is important to highlight that in women, no differences were found in 6-SM excretion among the three groups. This sexual dimorphism is difficult to explain; however, we cannot rule out a different effect of estrogen and androgen replacement therapy in GH deficiency, as most of our patients were receiving exogenous sex steroids. It is important to point out that the four menopausal women included in the treated group had appropriate values of estradiol according to healthy menopausal population, although not being substituted with exogenous estrogens. As regards to the not substituted hypogonadic male patient, his testosterone levels were slightly below the normal range. For that reason, we believe that it is unlikely that the absence of sex steroids replacement in these patients could influence the results. It is worth mentioning that oral estrogen is known to blunt the effect of GH replacement on hepatic tissue. Nevertheless our male and female treated patients reached non significant different levels of IGF-1, due to higher significant GH doses required in females. Interestingly, estrogens have been shown to modify melatonin production “*in vitro*” and “*in vivo*” (26,27). All these factors might contribute to make potential nighttime-daytime differences in GHD and non-GHD women less evident. The number of subjects included in each group in the present study is not sufficient to reach definitive conclusions, but it is important to point out that sexual dimorphism has already been reported by other authors in preschool children (28) and, in a previous study, we have also observed a sexual dimorphism in obese adolescents, where males showed higher nocturnal levels of 6-SM than females (13).

The circadian rhythms of melatonin and body temperature are set to an earlier hour in women than in men, even when women and men maintain nearly identical and consistent bedtimes and wake times. Such finding of a sex difference in intrinsic circadian period are consistent with studies conducted in a number of nonhuman animals that have demonstrated that circadian period is shorter in females than in males. The shorter intrinsic circadian period described in women may be attributable, partly, to the higher circulating levels of estrogen in women (29). In addition, exposure to high estrogen at some point during development could alter the hypothalamic circadian pacemaker, leading to the sexual dimorphism that has been reported in suprachiasmatic nucleus structure (30). Despite the well- known described sexual dimorphism in GH-IGF-1 secretion, this may not account for the differences found, since in the conditions of our study, all patients were treated with GH doses to maintain IGF-1 between 0 and +2 SDS. Under the conditions of this study, we also found differences in 6-SM levels between treated and untreated GHD patients in nighttime-daytime delta values of males, while in females no significant differences in 6-SM were found between treated and untreated GHD patients.

For many years, IGF-1 measurement has been used both in children and adults as a surrogate for GH secretion and it has even been used for monitoring exogenous GH therapy in different conditions, as IGF-1 concentrations remain stable during daytime, with diurnal variations not exceeding 25%. Therefore, we thought it would be interesting to correlate IGF-1 levels with melatonin levels in patients on and off GH replacement therapy. In a previous paper in male children and adolescents we found no correlations between 6-SM and IGF-1. The positive correlations found in adults in the present study could be explained by differences in melatonin and IGF-1 secretion at different ages, as well as by different relationships between melatonin production and peripheral signals in childhood and adulthood. Concerning the correlations observed in this study, a sexual dimorphism was observed. In males, we found a significantly positive correlations for total, nocturnal 6-SM levels and nighttime-daytime delta values and IGF-1 levels, whereas in females a significant correlations for total 6-SM and a tendency towards a statistical significance for diurnal 6-SM and IGF1 were observed. These facts, in an indirect manner, might suggest that there is a different impact between males and females on the effect of GH replacement over the circadian rhythm of melatonin secretion. This phenomenon could be possibly explained by the role of estrogens. It could also be related to a well-known physiological pattern of GH, which is released largely at night in complex ultradian pulses that do not readily follow a classic circadian pattern (10). Indirectly, the clock system directs secretion through slow-wave sleep which is strongly associated with GH release, although the precise mechanism of circadian influence is not understood. There is a sexual dimorphism of plasma GH with distinct male and female patterns of growth, which may be clock-determined (1). The evaluation of secretory pulses of serum melatonin during sleep might possibly allow us to elucidate these discrepancies. Such evaluation would obviously require a supplementary experimental design, including multiple blood samples all over the night in order to study melatonin nocturnal peaks (1,28).

A potential limitation of our study is that as groups were not paired; small differences might not achieve statistical significance. Besides, we cannot rule out the influence of etiology, duration of GH deficiency, age of onset, etc. Regarding GH treatment and melatonin secretion, it should also be taken into account that the administration of exogenous GH replacement therapy is not performed strictly following the rhythm of physiological secretion of this hormone, which occurs mainly during the night with multiple peaks occurring approximately 90 minutes after the onset of sleep (stages 3 and 4 non-REM sleep). Based on the above, we could hypothesize that, perhaps, GH treatment might allow for the correction of associated metabolic disorders in patients, but it might not be enough to resolve in all cases the abnormalities in the secretory pattern of melatonin occurring in these patients (31). The present study was not designed to correlate 6-SM with other parameters, such as body composition, lipid and glucose profile, sleep pattern or quality of life. Therefore, we could not draw conclusions regarding the clinical implications of melatonin alteration and metabolic impact, sleep characteristics or quality of life in AGHD patients.

An original contribution of this study has been the physiological and noninvasive evaluation of melatonin secretion dynamics, by measuring nocturnal and diurnal urinary excretion of 6-SM, in GH-deficient subjects on- and off-replacement therapy. Even if a potential methodological limitation of our study is the fact that the cross-sectional designed allowed us to evaluate correlations but not causality, GH-deficient patients showed lower levels of 6-SM. In order to confirm normalization of melatonin secretion with GH replacement therapy, a paired longitudinal study should be performed in patients prior to and during GH replacement therapy.

In conclusion, our findings provide an interesting approach to a further understanding of some chronobiological disorders involved in GH deficiency. Furthermore, they might also contribute to an interpretation of the pathophysiologic mechanism underlying some GHD alterations associated with quality of life and sleep-dependent metabolic disorders, which would put a new perspective to the design of future studies.
